# Use of the cardiopulmonary coupling index based on refined composite multiscale entropy for prognostication of acute type A aortic dissection

**DOI:** 10.3389/fcvm.2023.1126889

**Published:** 2023-03-08

**Authors:** Zhi-Jie Mao, Wei-Wei Wen, Yi-Chen Han, Wei-hua Dong, Li-juan Shen, Zhou-Qing Huang, Qiang-Li Xie

**Affiliations:** ^1^ The Key Laboratory of Cardiovascular Disease of Wenzhou, Department of Cardiology, The First Affiliated Hospital of Wenzhou Medical University, Wenzhou, China; ^2^ Department of Cardiovascular Care Unit, The First Affiliated Hospital of Wenzhou Medical University, Wenzhou, China

**Keywords:** cardiopulmonary coupling, acute type a aortic dissection, prognostication, composite multiscale entropy, risk stratification

## Abstract

**Objectives:**

The aim of this study is to assess the influence of cardiopulmonary coupling (CPC) based on RCMSE on the prediction of complications and death in patients with acute type A aortic dissection (ATAAD).

**Background:**

The cardiopulmonary system may be nonlinearly regulated, and its coupling relationship with postoperative risk stratification in ATAAD patients has not been studied.

**Methods:**

This study was a single-center, prospective cohort study (ChiCTR1800018319). We enrolled 39 patients with ATAAD. The outcomes were in-hospital complications and all-cause readmission or death at 2 years.

**Results:**

Of the 39 participants, 16 (41.0%) developed complications in the hospital, and 15 (38.5%) died or were readmitted to the hospital during the two-year follow-up. When CPC-RCMSE was used to predict in-hospital complications in ATAAD patients, the AUC was 0.853 (*p* < 0.001). When CPC-RCMSE was used to predict all-cause readmission or death at 2 years, the AUC was 0.731 (*p* < 0.05). After adjusting for age, sex, ventilator support (days), and special care time (days), CPC-RCMSE remained an independent predictor of in-hospital complications in patients with ATAAD [adjusted OR: 0.8 (95% CI, 0.68–0.94)].

**Conclusion:**

CPC-RCMSE was an independent predictor of in-hospital complications and all-cause readmission or death in patients with ATAAD.

## 1. Introduction

Acute type A aortic dissection (ATAAD) is a cardiovascular emergency disease that can kill 1%–2% of untreated patients every hour following the onset of symptoms (1). Despite timely emergency and essential surgery in the hospital, the in-hospital mortality and complications of patients with acute aortic dissection are still high. Fortunately, thanks to advances in surgical technology and improvements in perioperative care, mortality has decreased over the past decade to 12% (2). Therefore, it is necessary to improve risk prediction for patients with a high risk of complications and death to make timely and appropriate interventions.

The concept of cardiopulmonary coupling (CPC) was advocated in 2005 (3). CPC is now a technique that can reflect the function of cardiopulmonary system action relation and coupling strength by calculating the cross-spectral power and coherence of respiratory tidal volume fluctuations and heart rate variability (HRV) (4). It may provide information on the quantitative presentation of a person's manifestations of cardiovascular autonomic nervous function.

Since the cardiopulmonary system may be regulated in a nonlinear way, a new coupling analysis technique is needed. In recent years, the multiscale entropy (MSE) algorithm has gained massive attention, especially in the cardiovascular and physiological fields (5). Norris et al. found that heart rate (HR) MSE within hours of admission predicted mortality occurring later in 3,154 trauma patients (6). Many studies have shown that MSE is a method that can be used to generate novel clinical prognostic biomarkers.

No known research has focused on exploring the relationship between the definition of CPC based on MSE and the complications and deaths of patients with ATAAD. Therefore, we propose the following hypotheses: the cardiopulmonary coupling index based on refined composite multiscale entropy (CPC-RCMSE) can be used for successful risk stratification in ATAAD patients.

## 2. Methods

### 2.1. Study design

This was a prospective cohort study involving all patients after aortic dissection at the First Affiliated Hospital of Wenzhou Medical University from September 2018 through September 2020 (ChiCTR1800018319). The study protocol was approved by the Ethics Committee of the First Affiliated Hospital of Wenzhou Medical University.

The inclusion criteria were as follows: (1) patients aged 18–65 years; (2) patients who met the diagnostic criteria for acute type A aortic dissection and underwent surgery; and (3) patients or family members who voluntarily participated in the trial and signed informed consent. The exclusion criteria were as follows: (1) patients with chest and back deformity (including appearance and organic); (2) pacing patients; (3) patients with an ECG signal quality not up to standard; (4) patients in the terminal stage of chronic wasting disease; (5) patients in persistent coma; and (6) pregnant or nursing women.

### 2.2. CPC-RCMSE definitions

Electrocardiograph signals and respiratory waveforms were collected for more than 4 h on the first day after returning to the cardiac care unit. The CPC-RCMSE formula was described previously (7).

### 2.3. Primary outcome

The main end point was the occurrence of death, acute liver failure, acute renal failure, or ventilator-associated pneumonia composite end events in the hospital. The secondary endpoint was all-cause readmission or death at 2 years.

#### 2.3.1. Statistical analysis

Continuous variables are expressed as the mean ± standard deviation (SD). Categorical variables are expressed as the number (percentage). Groups of continuous variables were compared using Student's *t* test or the Mann‒Whitney *U* test, and groups of categorical variables were compared using the *χ*^2^ test or Fisher's exact test.

Univariate logistic regression analysis was used to investigate the independent risk factors for the compound endpoint of complications in the hospital in patients with ATAAD. All variables significantly associated with in-hospital mortality were candidate variables in the stepwise multivariate analysis. Based on the results of the multivariate logistic regression analysis, we explored whether CPC-RCMSE is an independent predictor of complications in the hospital. A receiver operating characteristic (ROC) curve for CPC-RCMSE was generated. Survival curves were described by the Kaplan‒Meier method and compared by the log-rank test. Statistical analyses were performed by SPSS 25.0 statistical software (SPSS Company, Chicago, IL) and the R language tool. In all analyses, *p* < 0.05 was statistically significant.

## 3. Results

### 3.1. Baseline characteristics

In this study, patients with aortic dissection were selected. The results are presented in Table 1. The average age of the study population was 53.6 ± 10.4 years. A total of 84.6% of the participants were male, 69.2% had hypertension, 16 (41.0%) developed complications in the hospital, and 15 (38.5%) died or were readmitted to the hospital during the two-year follow-up.

**Table 1:** Baseline characteristics of aortic dissection patients.

Variable	CPC-RCMSE			*p*-value
	T1 < 19.71 (*n* = 13)	T2 ≥ 20.69 to <29.91 (*n* = 13)	T3 ≥ 30.03 to <37.35 (*n* = 13)	
Age	56.15 ± 9.44	52.85 ± 9.93	51.77 ± 11.89	0.544
Male	11 (84.62%)	12 (92.31%)	10 (76.92%)	0.855
Hypertension	9 (69.23%)	10 (76.92%)	8 (61.54%)	0.697
Diabetes	0 (0.00%)	1 (7.69%)	0 (0.00%)	1.000
Smoking	5 (38.46%)	7 (53.85%)	4 (30.77%)	0.476
Drinking	5 (38.46%)	6 (46.15%)	3 (23.08%)	0.458
Coronary heart disease	1 (8.33%)	1 (7.69%)	0 (0.00%)	0.760
Chest pain	10 (76.92%)	10 (76.92%)	11 (84.62%)	1.000
Back pain	7 (53.85%)	7 (53.85%)	9 (69.23%)	0.654
Osphyalgia	5 (38.46%)	3 (23.08%)	3 (23.08%)	0.603
Abdominal pain	3 (23.08%)	1 (7.69%)	0 (0.00%)	0.297
Ventilator support (days)	140.08 ± 143.19	71.69 ± 73.48	51.15 ± 35.40	0.102
Extracorporeal circulation time (min)	258.08 ± 41.32	270.85 ± 30.57	264.31 ± 61.60	0.579
Aortic cross clamp time (min)	158.46 ± 43.37	172.46 ± 37.68	182.54 ± 52.91	0.460
Admission SBP	132.62 ± 24.23	136.69 ± 17.68	141.69 ± 23.99	0.702
Admission DBP	71.23 ± 13.91	71.54 ± 21.66	74.38 ± 17.93	0.852
Postoperative SBP	130.31 ± 31.14	129.54 ± 26.77	139.00 ± 22.91	0.643
Postoperative DBP	70.08 ± 14.82	69.54 ± 10.44	72.69 ± 13.16	0.842
Postoperative mean arterial pressure	92.54 ± 21.22	89.08 ± 16.24	95.69 ± 16.47	0.653
LVEF (%)	61.55 ± 7.31	64.94 ± 5.82	63.73 ± 5.59	0.332
Hypothermic circulatory arrest (min)	26.69 ± 3.61	29.08 ± 7.01	29.54 ± 5.01	0.380
Special care time (days)	11.00 ± 8.42	7.92 ± 4.52	7.62 ± 4.43	0.501
Preoperative heart failure	1 (7.69%)	0 (0.00%)	1 (7.69%)	1.000
Preoperative pleural effusion	1 (7.69%)	4 (30.77%)	1 (7.69%)	0.321
Preoperative pericardial effusion	3 (23.08%)	2 (15.38%)	1 (7.69%)	0.855
Norepinephrine/dopamine	4 (30.77%)	3 (23.08%)	1 (7.69%)	0.477
Vascular Drug	8 (61.54%)	8 (61.54%)	8 (61.54%)	1.000
ACE inhibitor	0 (0.00%)	0 (0.00%)	1 (7.69%)	1.000
Calcium channel blocker	3 (23.08%)	4 (30.77%)	3 (23.08%)	0.874
Beta blocker	2 (15.38%)	4 (30.77%)	2 (15.38%)	0.689
Postoperative pericardial effusion				0.648
0	12 (92.31%)	10 (76.92%)	9 (69.23%)	
1	1 (7.69%)	2 (15.38%)	2 (15.38%)	
2	0 (0.00%)	1 (7.69%)	2 (15.38%)	
Postoperative pleural effusion				0.081
0	2 (15.38%)	0 (0.00%)	0 (0.00%)	
1	4 (30.77%)	8 (61.54%)	3 (23.08%)	
2	7 (53.85%)	5 (38.46%)	10 (76.92%)	
Hospital days	24.23 ± 7.58	21.31 ± 9.06	21.46 ± 7.42	0.267
In-hospital complications	11 (84.62%)	3 (23.08%)	2 (15.38%)	<0.001
Death	2 (15.38%)	1 (7.69%)	0 (0.00%)	0.760
Readmission	9 (69.23%)	2 (15.38%)	3 (23.08%)	0.008
Cardiac readmission	5 (38.46%)	0 (0.00%)	0 (0.00%)	0.003
All-cause readmissions and deaths	9 (69.23%)	3 (23.08%)	3 (23.08%)	0.020

LVEF, left ventricular ejection fraction; Vascular Drug, sodium nitroprusside/urapidil/lyceryl trinitrate; In-hospital complications included acute renal failure, acute liver failure, respiratory failure, pulmonary infection and death.

#### 3.1.1. Independent prognostic factors

The univariate logistic analysis results are shown in Table 2. According to multivariate analysis, CPC-RCMSE was independently associated with in-hospital complications. After adjusting for age, sex, ventilator support (days), and special care time (days), CPC-RCMSE remained an independent predictor of in-hospital complications in patients with ATAAD [adjusted OR: 0.8 (95% CI, 0.68–0.94)]. In Model 2, CPC-RCMSE remained an independent risk factor for in-hospital complications after adjusting for age, sex, ventilator duration, special care time, cardioactive drugs, and vasoactive drugs [adjusted OR: 0.77 (95% CI, 0.63–0.95), *p* < 0.05].

**Table 2:** Association of CPC-RCMSE with in-hospital complications.

Clinical variables	Univariate analysis		Model 1		Model 2	
	Odds ratio (95% CI)	*p*-value	Odds ratio (95% CI)	*p*-value	Odds ratio (95% CI)	*p*-value
Age	1.04 (0.98–1.11)	0.19	0.98 (0.85–1.13)	0.79	0.98 (0.86, 1.12)	0.79
Male	0.10 (0.01–0.96)	<0.05	0.08 (0.00–4.32)	0.21	0.04 (0.00, 20.03)	0.30
Hypertension	0.96 (0.24–3.83)	0.96	–	–	–	–
Smoking	0.49 (0.13–1.89)	0.30	–	–	–	–
Drinking	0.71 (0.18–2.72)	0.61	–	–	–	–
Chest pain	0.63 (0.13–3.01)	0.56	–	–	–	–
Back pain	0.53 (0.14–1.96)	0.34	–	–	–	–
Osphyalgia	2.16 (0.52–8.90)	0.29	–	–	–	–
Abdominal pain	5.08 (0.48–54.03)	0.18	–	–	–	–
Ventilator support (days)^†^	5.10 (1.72–15.11)	<0.05	3.40 (0.67–17.22)	0.14	4.31 (0.61, 30.41)	0.14
Extracorporeal circulation time^†^	2.17 (0.06–82.94)	0.68	–	–	–	–
Aortic cross clamp time^†^	0.14 (0.01–1.81)	0.13	–	–	–	–
Admission SBP	0.99 (0.96–1.02)	0.71	–	–	–	–
Admission DBP	0.99 (0.95–1.03)	0.65	–	–	–	–
Postoperative DBP	1.00 (0.95–1.06)	0.86	–	–	–	–
LVEF^†^	0.58 (0.00–312.26)	0.87	–	–	–	–
Hypothermic circulatory arrest^†^	0.03 (0.00–1.63)	0.09	–	–	–	–
Special care time (days)^†^	8.67 (1.76–42.67)	<0.05	8.11 (0.55–120.46)	0.13	9.64 (0.56,164.44)	0.12
Preoperative heart failure	1.47 (0.08–25.32)	0.79	–	–	–	–
Preoperative pleural effusion	0.24 (0.02–2.58)	0.21	–	–	–	–
Preoperative pericardial effusion	1.54 (0.27–8.82)	0.63	–	–	–	–
Norepinephrine/dopamine	1.58 (0.33–7.56)	0.56	–	–	0.09 (0.00, 6.93)	0.27
Vascular Drug	0.68 (0.18–2.54)	0.57	–	–	0.38 (0.02, 8.79)	0.55
Calcium channel blocker	1.64 (0.38–6.97)	0.50	–	–	–	–
Beta blocker	0.40 (0.07–2.33)	0.31	–	–	–	–
CPC-RCMSE	0.82 (0.73–0.92)	<0.05	0.80 (0.68–0.94)	<0.05	0.77 (0.63, 0.95)	<0.05

Abbreviations as in Tables 1. ^†^Log-transformed.

Model 1 was adjusted for age, Male, Ventilator support (days) †, Special care time (days) †,.

Model 2 was adjusted for age, Male, Ventilator support (days) †, Special care time (days) †, Vascular Drug, Norepinephrine/dopamine.

### 3.2. ROC curve

**Figure 1:**
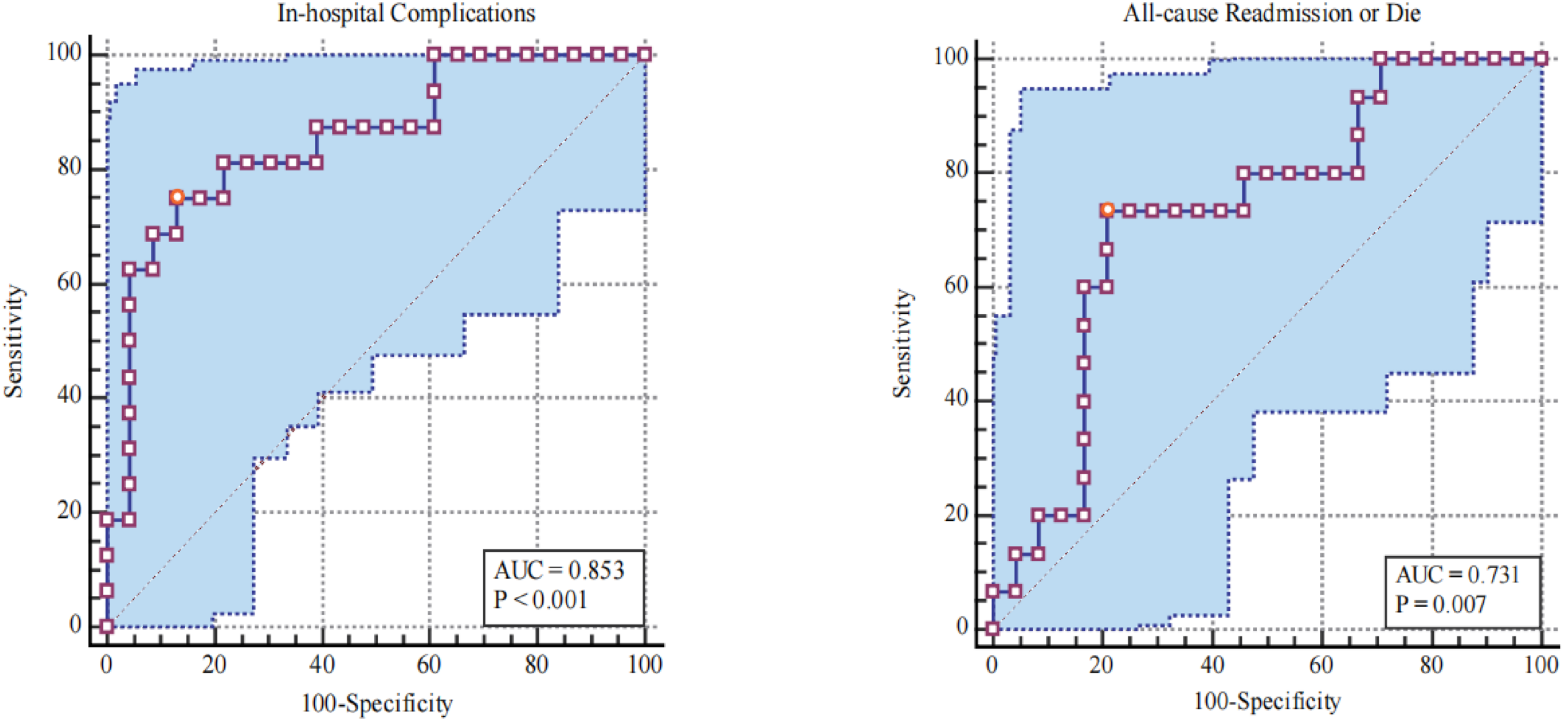
Receiving operating characteristic curves and corresponding AUCs for CPC-RCMSE.

As shown in Figure 1, when CPC-RCMSE was used to predict in-hospital complications in ATAAD patients, the AUC was 0.853 (*p* < 0.001). When CPC-RCMSE was used to predict all-cause readmission or death at 2 years, the AUC was 0.731 (*p* < 0.05).

### 3.3. Kaplan–Meier survival

**Figure 2:**
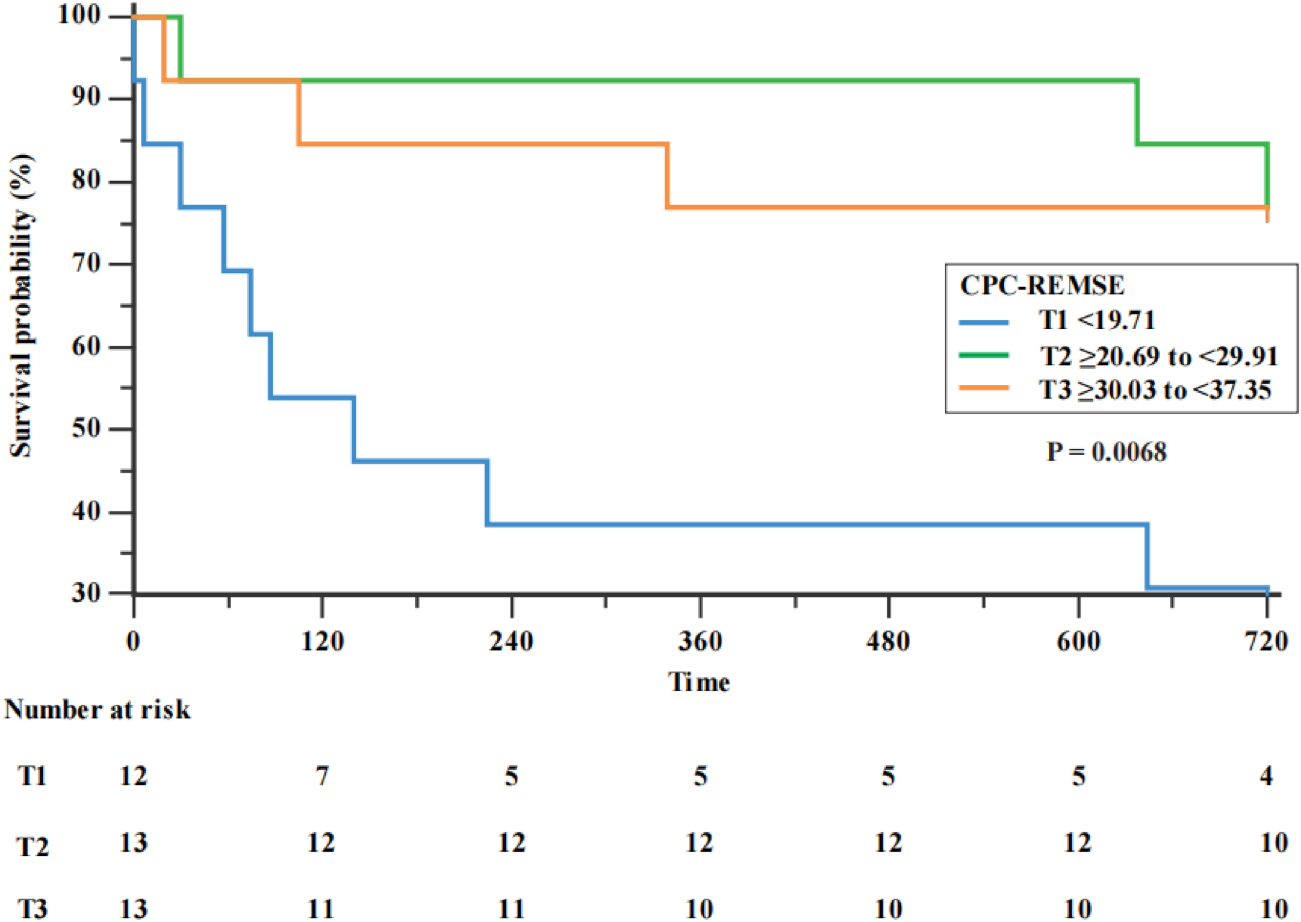
Kaplan–Meier survival by stage of CPC-RCMSE.

As shown in Figure 2, the Kaplan‒Meier curves showed significant survival outcomes for all-cause readmission or death at 2 years by CPC-RCMSE score. In patients with a higher CPC-RCMSE score, survival was better (*p* < 0.0001).

## 4. Discussion

In reviewing the literature, no data were found on the association between CPC-RCMSE and prognosis in ATAAD patients who have undergone surgery. Our findings highlight the prognostic importance for ATAAD. As expected, patients in the highest quintile of CPC-RCMSE had better outcomes. When CPC-RCMSE was used to predict the occurrence of postoperative complications in ATAAD patients, the AUC was 0.853 (*p* < 0.001), indicating that CPC-RCMSE had a good ability to predict the occurrence of complications in the hospital. When CPC complexity was ≤21.2, the sensitivity was 75%, and the specificity was 86.7%. When CPC complexity was used to predict readmission and death, the AUC was 0.731 (*p* < 0.05), also indicating good differentiation ability. The results of multivariate analysis indicate the clinical prediction and risk stratification value of CPC-RCMSE.

CPC (3) is based on continuous ECG signals and uses Fourier transform technology to analyze two characteristics of the signal: (1) heart rate variation and (2) the fluctuation of ECG R wave amplitude caused by respiration. From a physiological point of view, CPC can identify the bistable properties of automatic NREM sleep state transitions. One state shows deep sleep features, and the other state exhibits shallow sleep features. Many studies (8, 9) have reported its clinical significance, such as the 24-hour Holter-based CPC analysis (10), which showed that the recurrence rate was lower in atrial fibrillation patients who had unstable sleep before radiofrequency ablation (HR: 0.32; 95% CI, 0.12–0.83).

The cardiopulmonary system likely performs complex regulation in a nonlinear manner. The development of nonlinear dynamics and information theory has made new progress in the study of information transmission among multivariate time series and can complement traditional linear symmetry analysis technology to provide more diagnostic and prognostic information (11, 12). Costa et al. (13) proposed an MSE algorithm to calculate SampEn over a certain scale to represent the complexity of a time series. Pilatia et al. (14) described the application of the MSE algorithm to cardiopulmonary coupling. In our study, we used new algorithms. The RCMSE algorithm reduces the chance of generating indefinite entropy and is more suitable for the application of short time series analysis, such as physiological time series (7). Our study provide evidence for CPC-RCMSE on the prediction of complications and death in patients with ATAAD. Notably, many algorithms have proved to have advantages in the analysis of physiological signals in different study population. In terms of Congestive Heart Failure patients, Liu C et al. (15) determined the differential RR interval time series signal (MSE_dRR) had better statistical stability and better discrimination. Azami H. et al. (16) points out that Refined Composite Dispersion Entropy (RCMDE) increased ability to find differences between physiological signs better than RCMSE. However, the above algorithm has not been tested in ATAAD patients. For this reason, we expect compare the advantages and disadvantages of different algorithms in ATAAD patients with larger samples in future studies.

The results of our study indicated that CPC-RCMSE was an independent risk factor for ATAAD. ATAAD patients were divided into three groups according to their CPC-RCMSE (T1 < 19.71; T2 ≥ 20.69 to <29.91; T3 ≥ 30.03 to <37.35). The Kaplan‒Meier curves showed significant survival outcomes for all-cause readmission or death at 2 years by CPC-RCMSE score. In patients with lower CPC-RCMSE scores, survival was worse (*p* < 0.0001).

Additionally, we found that CPC-RCMSE was a strong independent predictor associated with in-hospital complications in our study population. When CPC complexity increased by 1 unit, the risk of hospital complications decreased by 20%. Clinical health care workers can be more targeted in meeting patient needs based on CPC-RCMSE scores to improve medical efficiency, shorten hospital stay, reduce care unit costs, and improve patient hospitalization outcomes.

## 5. Conclusions

CPC-RCMSE is an independent predictor of in-hospital complications in patients with ATAAD. CPC-RCMSE should be used more widely and routinely in the risk stratification of ATAAD patients.

## Data Availability

The original contributions presented in the study are included in the article/Supplementary Material, further inquiries can be directed to the corresponding author.
